# Fear and Coping in Students during the Early Stages of the COVID-19 Pandemic: A Combined Cross-Sectional and Longitudinal Study

**DOI:** 10.3390/ijerph18126551

**Published:** 2021-06-18

**Authors:** Anni M. Hasratian, Hannah O. Nordberg, Alicia E. Meuret, Thomas Ritz

**Affiliations:** Department of Psychology, Southern Methodist University, Dallas, TX 75206, USA; ahasratian@smu.edu (A.M.H.); hnordberg@smu.edu (H.O.N.); ameuret@smu.edu (A.E.M.)

**Keywords:** COVID-19 fears, RDoC Negative Valence Systems, coping, medical fears, depression

## Abstract

The overwhelming impacts of the COVID-19 pandemic have been experienced by individuals across the world. Additional circumstances unique to students affected their studies during the early stages of the pandemic, with changes in living and studying mid-semester. The current study aimed to investigate predictors of fear of COVID-19 in college students during this acute phase using cross-sectional and longitudinal samples. In total, 175 undergraduate students completed an online questionnaire in the spring 2020 semester following lockdown. A subset of 58 students completed a separate survey in fall 2019, which served as a baseline. For the cross-sectional sample, pre-COVID-19 and current living situations did not predict COVID-19 fears. However, a propensity to experience panic was significantly associated with greater COVID-19 fears. How students coped with the pandemic was not associated with COVID-19 fears, although a greater propensity to use denial as a coping style tended to be related to greater COVID-19 fears. In the longitudinal subsample, students showed decreased positive mood and social stress load while depressive mood increased after lockdown. Their preferred coping styles changed, utilizing more self-distraction and acceptance, and less self-blame and substance use. Findings reflect both positive and negative consequences of the pandemic. The unique changes in students’ lifestyles will need to be met by tailored interventions.

## 1. Introduction

The ongoing COVID-19 pandemic poses an unparalleled threat to human health and life. During the early stages of the pandemic, worldwide stay-at-home, lockdown, or quarantine orders isolated billions of people in their homes, with limited access to daily life resources and social interactions. Whereas the major focus of international efforts has been on saving lives, it is predicted that the mental health fall-out from this crisis will soon occupy professionals for years [1,2]. It is well-known that major negative life events can have profound influences on mental health [3]. The direct impact of the pandemic, and the drastic worldwide measures to combat it, have led to an accumulation of stress exposure at an unprecedented scale. Fear of infection, exposure to threatening and contradicting media information, confusion about appropriate health behaviors, isolation from social contacts, pronounced lifestyle limitations and changes in the home environment, including displacement, loss of occupation and income, witnessing illness and death of relatives and friends, and even combatting grave illness from the virus for oneself, all can be expected to elevate the risk for mental illness, particularly anxiety disorders and major depression.

Many sections of the population have been affected in both shared and unique ways by the COVID-19 pandemic. Whereas college students shared many of the challenges and restrictions experienced by the general population, they were also more likely to be affected by displacement. A few weeks into the unfolding pandemic, college students were faced with closing dormitories and campus facilities half-way through their studies, forcing them to relocate to friends or families, sometimes without full access to their personal belongings and previous social networks. Academic studies, challenging by themselves, had to be reorganized to online instruction, with limited access to instructors and learning resources. However, negative life events do not affect everyone equally [4]. Major catastrophes such as Hurricane Katrina exposed inhabitants of New Orleans and neighboring regions of the US Gulf Coast with some of the same consequences, including displacement, loss of property, and loss of social networks, and led to elevated levels of anxiety and depression in many. Notwithstanding, severe clinical sequelae such as trauma or suicide were seen only in a small proportion of the affected population [5]. History of mental illness is known to be a risk factor for particularly adverse reactions to life events [6]. However, studies of the psychological impact of major negative life events or disasters often lack adequate baseline data to gauge the impact of, and to explore predictors of differential psychological responding to, the event adequately. The study of such predictors is essential for the treatment and future prevention of psychological distress. Stress experience is also known to be associated with elevated infection risk [7], potentially initiating a vicious circle of infection and distress. Further, how one copes with negative life events can influence mental health outcomes (e.g., Sharma et al. [8]).

Finally, responses to negative life events can be examined using the Research Domain Criteria (RDoC) framework [9]. The RDoC initiative, developed by the National Institute of Mental Health, was devised to study mental illness under a dimensional perspective of multi-level neurobiological systems that include individuals’ experiences measured by psychometrically validated self-report instruments [10]. More specifically, the RDoC Negative Valence Systems demonstrates responses to adverse situations or context, including responses to acute threat, potential threat, sustained threat, frustrative non-reward, and loss [10]. Examining how Negative Valence Systems are affected during the early stages of the pandemic allows for further distinction between constructs, such as acute and sustained threat in response to the pandemic and loss as it relates to displacement.

The current study had three main objectives. The first aim was to examine levels of fear of viral infection with COVID-19 and cross-sectional associations with students’ life situations, coping styles, and RDoC Negative Valence Systems constructs following the early pandemic lockdown phase. Second, using the longitudinal subsample, we aimed to prospectively identify changes in key Negative Valence Systems measures and coping styles in response to the pandemic. This distinguishes our study from recent surveys that lacked baseline assessments and had to rely on retrospective reports of pre-pandemic mood levels and activities (e.g., Nikolaidis et al. [11]), which are prone to biases based on personality factors and current mood states. Finally, for the longitudinal subsample, we investigated baseline levels of medical fears and their potential to predict fear levels during the pandemic. Such prospective associations can provide information about individual characteristics that make students particularly vulnerable to excessive fear responses during viral pandemics.

## 2. Method

### 2.1. Participants and Procedures

Responses from 185 undergraduate students were collected between 20 April and 1 May 2020. Ten responses were removed because students consented but did not participate. The final sample size was 175, of whom 58 had also participated in a separate study in the fall semester (between August and December 2019), which served as a baseline for the current study. Students completed demographics, questions pertaining to their living situation prior to and following spring break, and eight self-report measures. The entire study was conducted online through Qualtrics, and students received course credit for participating. The Southern Methodist University Institutional Review Board approved this study and students provided written informed consent.

### 2.2. Measures

#### 2.2.1. Demographics and Life Situation Questions

Participants answered questions about demographic characteristics (e.g., race, ethnicity, age, and gender). Participants in spring 2020 also answered questions about life situations (e.g., living situations prior to and after lockdown) and provided information on whether they were tested or diagnosed for COVID-19.

#### 2.2.2. Fear of COVID-19

For participants in spring 2020, the seven-item Fear of COVID-19 Scale (FCV-19S) [12] was used to assess fear of COVID-19. Participants rated their level of agreement with each statement on a 5-point scale ranging from strongly disagree to strongly agree. Internal consistency for an English language version of the scale was shown to be α = 0.88 [13].

#### 2.2.3. RDoC Negative Valence Systems

The RDoC Negative Valence Systems are responses to aversive situations or context, such as fear, anxiety, and loss [10]. Although identification of self-report instruments that reflect the RDoC Negative Valence Systems has lagged behind system markers for behavioral and biological levels [14], we recently identified potential self-report markers of these systems using a larger pool of candidate questionnaire measures [15,16,17]. Therefore, six self-report measures were selected to represent the five negative valence domain constructs of acute threat (e.g., fear; panic), potential threat (e.g., worry; inhibition), sustained threat (e.g., chronic stress), frustrative non-reward, and loss. These questionnaires were administered both in fall 2019 and after the onset of the pandemic in spring 2020.

The Inventory of Depression and Anxiety Symptoms—Expanded Version (IDAS-II) [18] assesses eighteen specific symptoms of internalizing disorders, such as anxiety disorders, bipolar, and posttraumatic stress. These 99 items were rated on a five-point scale (1 = not at all to 5 = extremely). The IDAS-II subscales have demonstrated adequate internal consistency (α = 0.72–0.90) and validity [18].

The Behavioral Inhibition Scale, Behavioral Activation Scale, and Frustrative Non-Reward Scale (BIS (Behavioral Inhibition Scale)/BAS (Behavioral Activation Scale)/FNR(Frustrative Non-Reward Scale)) [19] is an extension of the BIS/BAS [20], which measures response to punishment and reward signals. The behavioral inhibition subscale includes 7 items (α = 0.74), and the behavioral activation system is measured in three subscales of 5-item reward responsiveness (α = 0.73), 4-item drive (α = 0.76), and 4-item fun seeking (α = 0.66) [20]. The 5-item FNR scale (α = 0.68) measures reduced approach motivation following non-reward [19]. Participants rated the items from 1 (very true for me) to 4 (very false for me).

The Penn State Worry Questionnaire (PSWQ) [21] assesses the trait of worry and is rated from 1 (not at all typical of me) to 5 (very typical of me). The PSWQ has demonstrated good internal consistency (α = 0.93) and good test–retest reliability (*r* = 0.92) over an 8–10-week period.

Facets of chronic stress were measured by the Trier Inventory of Chronic Stress (TICS) [22], where 57 items were rated from 0 (never) to 4 (always). The TICS contains nine subscales which have demonstrated good internal consistency (α = 0.84–0.91) [22].

The Positive and Negative Affect Schedule (PANAS) [23] includes ten positive affect and ten negative affect items. The PANAS was rated on a 5-point scale (1 = very slightly or not at all to 5 = extremely). The PANAS subscales have demonstrated good internal consistency (α = 0.86–0.90 for positive affect; α = 0.84–0.87 for negative affect) [23].

Finally, the 14-item Hospital Anxiety and Depression Scale (HADS) [24] assesses aspects of depressive and anxious mood. The items were rated between 0 and 3 with varying anchors depending on the item. The HADS subscales have demonstrated adequate internal consistency (α = 0.68–0.93 for HADS Anxiety; α = 0.67–0.90 for HADS Depression) [24]. The HADS Anxiety subscale (*r* = 0.89) and HADS Depression subscale (*r* = 0.86) have shown acceptable test–retest reliability over a three-week period [25].

#### 2.2.4. Coping

The Brief COPE [26] includes 14 scales covering a variety of coping styles rated from 0 (I haven’t been doing this at all) to 3 (I’ve been doing this a lot). The regular instruction for the Brief COPE was used for the longitudinal sample in fall 2019, but for administration in spring 2020, participants were prompted to answer based on the current situation of the virus pandemic. Internal reliability for the subscales has been shown to be acceptable, ranging from 0.50 to 0.90 [26].

#### 2.2.5. Medical Fears

The Medical Fear Survey—Short Version (MFS-short version) [27] was administered in the fall 2019 baseline study, where a subset of the sample (*n* = 58) completed the questionnaire. Of the five domains, we utilized the Examinations and Symptoms subscale (6 items). Items were rated from 0 (no fear or concern at all) to 3 (intense fear). The MFS-short Examinations and Symptoms subscale has demonstrated acceptable internal consistency (α = 0.76-0.84) [27].

### 2.3. Statistical Analyses

#### 2.3.1. Cross-Sectional Sample

SPSS Statistical Software version 25.0 (IBM Corporation, Armonk, NY, USA) was used for all data analyses. Descriptive statistics were computed for each measure in the datasets (fall pre-COVID-19 and spring COVID-19). Expectation maximization [28] was used to estimate missing values on measures that participants completed at least 25% of or more. Expectation maximization was conducted for the eight scales in the fall 2019 semester dataset and the eight scales in the spring 2020 semester dataset.

Multiple imputation was then used to estimate scale scores [29] for measures that participants did not complete or completed less than 25% of. Greatest percentage of missing data for a scale in each dataset determined the number of imputations. In the fall dataset, 11 imputations were included, and in the spring dataset, 7 imputations were included.

Using the multiple imputation datasets, hierarchical multiple linear regression models were used to predict fear of COVID-19. Control variables of age, gender, race, and ethnicity were entered in Step 1 for each of the three models, with living situations, RDoC Negative Valence Systems measures, or coping styles included in Step 2. The pooled results of the regression models were used. Semipartial $r^{2}$ (*r*_sp_^2^) estimates were calculated as estimates of the unique variance accounted for by each individual predictor in the pooled model. Since the pooled model in the regression does not provide R-Squared ($R^{2})$ estimates, total variance accounted for by the models was estimated using the original data.

#### 2.3.2. Longitudinal Subsample

Paired-sample *t*-tests were used with the baseline longitudinal subsample to investigate differences in RDoC Negative Valence Systems measures and coping styles from pre-COVID-19 (fall semester) to COVID-19 (spring semester) phases. A dataset containing measures that the subsample completed during both assessment timepoints was used for this analysis. Scores on the RDoC Negative Valence Systems construct scales and coping scales were compared between timepoints within participants in separate analyses. A hierarchical linear regression model was used to predict fear of COVID-19 by medical fears. Two additional exploratory hierarchical linear regression analyses were conducted with RDoC Negative Valence Systems measures and coping strategies that significantly differed between timepoints. Change scores for RDoC Negative Valence Systems measures and coping styles were calculated between timepoints, and these scores were included as predictors. Control variables on all models were age, gender, race, and ethnicity.

## 3. Results

### 3.1. Demographic and Life Situation Characteristics

Participants in the longitudinal subsample were predominately Caucasian (72.4%), Non-Hispanic (89.7%), and identified as female (74.1%). Mean age of participants was 19.67 (*SD* = 2.34) and ranged from 18 to 32 years of age.

Participants in the cross-sectional sample shared similar demographic characteristics and were predominately Caucasian (75.4%), Non-Hispanic (89.1%), and identified as female (66.9%). Their ages ranged from 18 to 32 years (*M* = 19.83, *SD* = 1.74). Participants’ school levels were generally distributed evenly, with slightly fewer students on upper class levels: 51 freshmen (29.1%), 57 sophomores (32.6%), 41 juniors (23.4%), and 26 seniors (14.9%). Eight participants (4.7%) reported that they were tested for COVID-19, and one participant (0.6%) reported that they had been diagnosed with COVID-19. Prior to lockdown, 71.8% of participants lived in on-campus housing, and 85.1% of participants reported that their living situation changed due to COVID-19 campus lockdown (e.g., moved out from on-campus or off-campus housing to living with family).

### 3.2. Fear of COVID-19

Students rated their average fear of COVID-19 as 15.33 (*SD* = 5.75) on a scale between 7 and 35. Item means ranged from 1.59 to 2.87. Internal consistency of the scale was α = 0.86.

Hierarchical multiple regression showed that COVID-19 fears did not vary with past and current life situations during the pandemic (Table 1).

### 3.3. RDoC Negative Valence Systems Markers as Correlates of Fear of COVID-19: Cross-Sectional Associations

The hierarchical multiple regression showed that 25.8% of the total variance in COVID-19 fears was explained by the predictor variables. Age, gender, race, and ethnicity accounted for 3.2% of the total variance, whereas 22.6% of the total variance in FCV-19S was explained by the inclusion of the RDoC negative valence measures. IDAS-II Panic (part of the acute threat construct) and gender were significant predictors of COVID-19 fears. Greater panic scores predicted greater COVID-19 fears, and fear of COVID-19 was greater for females compared to males (Table 2).

### 3.4. Coping Styles as Correlates of Fear of COVID-19: Cross-Sectional Associations

The hierarchical multiple regression showed that 18.4% of the total variance was explained by the independent variables. When controlling for demographics, which accounted for 2.9% of the total variance, the Brief COPE subscales explained an additional 15.5% of the total variance in FCV-19S. None of the coping styles alone were significant predictors of COVID-19 fears, although denial reached statistical trend levels (*p* = 0.056), indicating a trend towards greater denial predicting greater fear of COVID-19 (Table 3).

### 3.5. Change in RDoC Negative Valence Systems Constructs during the Pandemic: Longitudinal Analysis

Paired t-tests revealed significant differences in the loss and sustained threat constructs between the fall (pre-COVID-19) and spring (COVID-19) semesters. With regard to the loss construct, there was a significant decrease in PANAS Positive and a significant increase in HADS Depression. TICS Social Overload, part of the sustained threat construct, significantly decreased from fall to spring (Table 4).

### 3.6. Change in Coping Styles during the Pandemic: Longitudinal Analysis

Paired *t*-tests indicated significant differences in coping strategies between pre-COVID-19 and COVID-19 time points. There was an increase in self-distraction and acceptance and a decrease in substance use and self-blame at the COVID-19 assessment (Table 4).

### 3.7. Association of Changes in RDoC Negative Valence Systems Markers and Coping Styles with Fear of COVID-19: Exploratory Longitudinal Analysis

Follow-up hierarchical multiple regression analyses indicated that changes in RDoC negative valence markers of loss (HADS Depression, PANAS Positive) and sustained threat (TICS Social Overload) between timepoints were not significant predictors of COVID-19 fears. Changes in self-distraction, acceptance, substance use, and self-blame coping styles were also not significant predictors of COVID-19 fears.

### 3.8. Medical Fears as Longitudinal Predictors of Fear of COVID-19

The hierarchical multiple regression showed that 21.4% of the total variance was explained by demographic characteristics and MFS Examinations and Symptoms, with the MFS subscale accounting for 16.5%. MFS Examinations and Symptoms was a significant predictor of COVID-19 fears in the longitudinal group, with higher medical fears predicting greater COVID-19 fears.

## 4. Discussion

This study aimed to examine predictors of COVID-19 fears during the early pandemic lockdown period using cross-sectional and longitudinal samples. For the cross-sectional sample, pre-lockdown living conditions, changes in living situations due to COVID-19, and whether stay-at-home orders were enforced did not predict fear of COVID-19. However, a tendency to experience panic was found to be associated with greater COVID-19 fears. In addition, coping styles were not associated with fear of COVID-19, although a greater propensity to use denial tended to be associated with greater COVID-19 fears. In the longitudinal analysis, it was of particular interest that positive mood declined and depressive mood increased in students after lockdown, while, at the same time, social stress load decreased, pointing to both positive and negative consequences of the pandemic for students. Changes in preferred coping styles also occurred, as students utilized more self-distraction, reduced self-blame and substance use, and enhanced their ability to accept adverse circumstances, again reflecting both positive and negative consequences of the pandemic and the associated change in living and studying situation. Whereas longitudinal changes in none of the negative valence markers or coping styles predicted COVID-19 fears, the more specific aspect of medical fears, the fear of examinations and symptoms, predicted greater COVID-19 fears as expected.

The overall level of COVID-19 fear was modest in our sample but comparable to a British community sample [13], as compared to greater fears reported from the Iranian original [12]. Our findings suggest that higher panic symptoms may lead to more heightened fears of viral infection during the pandemic. Previous research has shown a relation between stressful life events and the onset of panic symptoms (see Klauke et al. [30] for review). Panic is characteristically subsumed under the RDoC acute threat construct, which is defined as the activation of the brain’s defensive motivational system in order to promote behaviors that protect individuals from perceived danger [10]. Harper and colleagues [13] found that fear of COVID-19 was a predictor of positive behavior changes, such as maintaining social distancing and improving hand-washing hygiene.

Although only visible at the trend level, it was interesting that coping by denial was associated with higher COVID-19 fears. A prospective study examining anxiety and coping responses to the 2003 SARS epidemic found that avoidance coping strategies predicted more state anxiety [31]. Denial is an avoidant coping strategy, suggesting that the trend found in our sample is similar to what has been found in the literature. Another study investigating the psychological and coping responses to the SARS outbreak reported an association between posttraumatic morbidity and increased use of denial as a coping style [32]. Similar to the current study, Sim et al. [32] also used the Brief COPE to assess coping styles. With regard to the effects of coping styles during the COVID-19 pandemic, Yu et al. [33] examined the psychological states of citizens of China during the early stages of the pandemic, finding that greater use of passive coping styles (e.g., refusal to acknowledge existence of stressful events) predicted higher psychological distress. Our findings could suggest that avoidant or passive coping, such as denial, could also lead to greater fear of viral infection. However, this interpretation has to be made with caution, given that the finding did not reach conventional levels of statistical significance.

Findings furthermore showed a significant decrease in positive affect and increase in depressive symptoms from pre-COVID-19 to COVID-19 timepoints on markers of the RDoC Negative Valence Systems construct of loss. Both aspects could indicate changes in the loss system, although the exact conceptualization of this negative valence construct and its overlap with Positive Valence System constructs requires further study [14]. It is important to note that although depressive symptoms increased, the scores remained within non-clinical levels of depressive symptoms. Our findings reflect the results of a student survey in Switzerland, which showed increases in depressive symptoms in students under lockdown conditions that were also within non-clinical levels [34]. A change in the RDoC loss construct aligns with students’ experience of displacement (i.e., having to leave on-campus housing) mid-semester. Students of the Swiss survey also reported increases in anxiety, perceived stress, and loneliness, which could be due to more severe restrictions on daily life or a less cohesive social campus network compared to US colleges. In support of the latter, one potential marker of the RDoC sustained threat construct, social stress overload, decreased in our sample during the COVID-19 crisis. It is interesting that the termination of campus life and displacement apparently had the positive effect of students feeling reduced pressure to be socially active, a pressure that could, at least for some, be the negative consequence of campus culture and developmental stage in life that emphasizes social activities characterized by intensive interaction with peers in addition to academic strive.

In addition to changes in these RDoC constructs, there was an increase in the coping styles of self-distraction and acceptance and a decrease in self-blame. Emerging research also suggests both an overall increase in substance use during the pandemic [35,36] and increased substance use to cope with stress or emotions specifically related to COVID-19 [37]. Likewise, Hawke and colleagues [38] reported that youths in a clinical group comprising participants from mental health and substance use treatment programs reported greater substance use as a coping strategy compared to community participants. By contrast, our findings show decreased substance use as a form of coping (general substance use was not assessed). It is possible that the change in living situation, from relatively liberal campus life to a more supervised family home setting, had the positive consequence of reducing substance use as a way of coping with daily life challenges in this population.

This study is limited by its relatively small sample size, which did not allow for more predictor variables and examination of interaction effects. This also limited our ability to conduct additional analyses with the longitudinal subsample, which could have provided additional insight into prospective individual differences in students during the acute phase of the lockdown. Future research should aim to address this limitation by recruiting a larger sample of students (e.g., potentially recruiting graduate students in addition to undergraduates) and including additional study timepoints to allow for the examination of more fine-grained temporal relations between variables. The lower-than-expected recruitment may have been in part because of the semester interruption due to COVID-19. Additionally, with end-of-semester final examinations impending in an unfamiliar format (i.e., virtually), students may have been less active in seeking research participation. However, there was a fairly equal representation of school year levels within the sample. Therefore, our findings were not attributable to year-specific stressors (e.g., newly adjusting freshmen or graduating seniors), which allows for generalizability across student populations.

A second limitation is that the samples were predominately female, Non-Hispanic, and Caucasian. These demographics do not represent college students in the USA as a whole [39] but reflected the university’s diversity profile at large. Undergraduate students who identify as Asian or Black or African American currently constitute 13.1% of the undergraduate population [40] and 20.7% of participants in the longitudinal subgroup, and 21.1% of participants in the cross-sectional sample identified as Asian, or Black or African American. Further, gender, ethnicity, and race were controlled in all analyses, raising confidence in the interpretation of our findings.

Different instructions for the Brief COPE during the fall and spring semesters might also be considered a limitation. Although students in the spring 2020 study were prompted to answer the questions based on the current situation of the virus pandemic, one could argue that their responses would have reflected their coping styles in response to the pandemic regardless of the specificity of the instructions. The questionnaire time frame remained the same for both the fall and spring semesters; therefore, the current pandemic would likely be captured in the responses during the spring semester.

Despite these limitations, the current study has several strengths. First, using fall 2019 data as a baseline, we were able to prospectively study predictors of COVID-19 fears. By collecting data at two timepoints, with a baseline in the fall 2019 semester, we eliminated the prospect of recall bias, which retrospective studies typically encounter. This allowed us to investigate the psychological impact of COVID-19 on students longitudinally and examine changes in negative affect and coping styles during the early stages of the pandemic. Second, the use of instruments that closely track the National Institute of Mental Health RDoC conceptualization of Negative Valence Systems [15,16,17] enables alignment with the emerging literature that seeks to develop an understanding of disruption of mental health based on a dimensional model of multilevel systems that are anchored in biological processes [9,41]. Third, conducting the study after students experienced displacement allowed for the examination of how changes in living situations were associated with COVID-19 fears.

With the psychological impacts of COVID-19 already apparent, it is critical for the field to identify interventions that address these hardships. Knowing what factors contribute to greater fears of this viral infection can help to identify individuals who may benefit in particular from additional psychological assistance. This can allow for the use of individualized treatments. For instance, treatments aimed at symptom reduction, such as panic, could be helpful in reducing increased emotional distress in students with a propensity to experience panic. Moreover, skills training can be utilized to reduce avoidance coping and increase the use of adaptive coping strategies, which can help to build resilience.

## 5. Conclusions

While the negative impacts of the COVID-19 pandemic have been experienced globally, students were faced with additional stressors unique to the college experience. During the early stages of the pandemic, lockdown and stay-at-home measures forced students to relocate mid-semester and resume their studies virtually. However, these drastic changes in living situations did not predict greater fears of COVID-19, nor did coping styles in response to the pandemic. The greatest predictor of COVID-19 fears in students was the tendency to experience more panic. Further, in addition to negative consequences, such as decreased positive mood and increased depressive mood, positive consequences also existed, as demonstrated by the longitudinal subsample. Reduced self-blame and substance use as preferred coping styles enhanced students’ ability to adapt to and accept adverse circumstances. These findings highlight the importance of coping skills in managing distress during the COVID-19 pandemic, and the propensity to experience panic as an anxiety symptom related to greater COVID-19 fear in students. The present study provides support for tailored treatments, such as targeting panic, as well as monitoring mood (i.e., decrease in positive affect and increase in negative affect) in response to COVID-19 fears. It also emphasizes adaptive coping skills that can help to reduce distress (e.g., less self-blame).

Although these results reflect students’ responses during the early lockdown stages, college students continue to be uniquely affected by the pandemic, with the college experience continuing to look different for many throughout the different waves of the pandemic. Therefore, future studies should continue to examine the long-term psychological impacts of disrupted learning, changes in social networks, and navigation of the many challenges of the pandemic with the aim of developing effective interventions that preserve or restore the productivity, mental health, and quality of life of the college student population.

## Figures and Tables

**Table 1:** Hierarchical multiple linear regression analysis results of living situations predicting fear of COVID-19.

Predictor	*B*	SE	t	*p*	*r*_sp_^2^
Age	−0.48	0.26	−1.84	0.066 ^†^	0.020
Gender (0, male; 1, female)	−1.23	0.94	−1.31	0.191	0.010
Race (0, Caucasian; 1, Other)	0.48	1.11	0.43	0.664	0.001
Ethnicity (0, Non-Hispanic; 1, Hispanic)	−0.72	1.63	−0.44	0.658	0.001
Living situation prior to spring break (0, on campus; 1, off-campus)	1.35	1.27	1.06	0.288	0.007
Living situation changed due to COVID-19 regulations (0, yes; 1, no)	−1.41	1.63	−0.87	0.385	0.005
Stay-at-home orders enforced (0, yes; 1, no)	−0.71	1.41	−0.50	0.616	0.002

*Note*. *Abbreviations*: *r*_sp_^2^, semipartial *r*^2^. ^†^ *p* < 0.10.

**Table 2:** Hierarchical multiple linear regression analysis results of the Research Domain Criteria Negative Valence Systems construct markers predicting fear of COVID-19.

Predictor	*B*	SE	t	*p*	*r*_sp_^2^
Age	−0.36	0.24	−1.51	0.131	0.011
Gender (0, male; 1, female)	−1.76	0.88	−2.00	0.046 *	0.019
Race (0, Caucasian; 1, Other)	0.65	1.01	0.65	0.518	0.002
Ethnicity (0, Non-Hispanic; 1, Hispanic)	−0.81	1.58	−0.51	0.611	0.001
IDAS-II Panic	0.29	0.12	2.40	0.018 *	0.035
IDAS-II Claustrophobia	0.32	0.18	1.80	0.073 ^†^	0.016
IDAS-II Traumatic Avoidance	0.22	0.18	1.21	0.230	0.008
BIS	0.28	0.15	1.89	0.060 ^†^	0.020
PSWQ	0.07	0.07	0.97	0.334	0.005
TICS Excessive Demands at Work	−0.01	0.25	−0.04	0.972	<0.001
TICS Social Overload	−0.11	0.17	−0.65	0.516	0.002
TICS Work Overload	−0.15	0.18	−0.85	0.404	0.007
HADS Depression	−0.16	0.16	−1.04	0.300	0.005
PANAS Positive	0.00	0.06	0.01	0.992	<0.001
FNR	−0.07	0.16	−0.45	0.656	0.001

*Note*. *Abbreviations*: *r*_sp_^2^, semipartial *r*^2^. ^†^ *p* < 0.10, * *p* < 0.05. IDAS-II, Inventory of Depression and Anxiety Symptoms-Expanded Version. BIS, Behavioral Inhibition Scale. PSWQ, Penn State Worry Questionnaire. TICS, Trier Inventory of Chronic Stress. HADS, Hospital Anxiety and Depression Scale. PANAS, Positive and Negative Affect Schedule. FNR, Frustrative Non-Reward.

**Table 3:** Hierarchical multiple linear regression analysis results of coping styles predicting fear of COVID-19.

Predictor	*B*	SE	t	*p*	*r*_sp_^2^
Age	−0.27	0.26	−1.04	0.299	0.006
Gender (0, male; 1, female)	−0.32	0.95	−0.34	0.736	0.001
Race (0, Caucasian; 1, Other)	0.90	1.12	0.80	0.423	0.004
Ethnicity (0, Non-Hispanic; 1, Hispanic)	−0.90	1.69	−0.54	0.593	0.002
Self-distraction	0.69	0.46	1.48	0.142	0.015
Active coping	0.02	0.58	0.04	0.968	<0.001
Denial	1.71	0.89	1.92	0.056 ^†^	0.023
Substance use	0.48	0.64	0.74	0.459	0.003
Emotional support	0.97	0.57	1.72	0.086 ^†^	0.017
Instrumental support	0.80	0.65	1.23	0.218	0.009
Behavioral disengagement	−0.39	0.76	−0.51	0.609	0.002
Venting	−0.59	0.62	−0.95	0.344	0.006
Positive reframe	−0.33	0.45	−0.72	0.471	0.003
Planning	−0.35	0.54	−0.65	0.515	0.003
Humor	0.20	0.42	0.49	0.626	0.001
Acceptance	−0.71	0.41	−1.72	0.086 ^†^	0.017
Religion	−0.26	0.41	−0.63	0.529	0.002
Self-blame	−0.13	0.68	−0.19	0.849	<0.001

*Note*. *Abbreviations*: *r*_sp_^2^, semipartial *r*^2^. ^†^ *p* < 0.10.

**Table 4:** Change in proposed Research Domain Criteria Negative Valence Systems construct measures and coping strategies during the pandemic.

Outcome	Fall 2019	Spring 2020		95% CI for Mean Difference			
	(*M*, *SD*)	(*M*, *SD*)	n		r	t	df
IDAS-II Panic	12.44 (5.43)	12.19 (5.51)	54	−0.90, 1.41	0.70 ***	0.45	53
IDAS-II Claustrophobia	6.80 (3.16)	6.35 (3.04)	54	−0.57, 1.46	0.28 *	0.88	53
IDAS-II Traumatic Avoidance	7.54 (3.55)	6.91 (3.20)	54	-0.31, 1.57	0.48 ***	1.34	53
BIS	21.00 (3.23)	21.43 (3.30)	53	−1.26, 0.39	0.58 ***	−1.05	52
PSWQ	32.17 (7.23)	32.43 (7.69)	53	−1.90, 1.37	0.69 ***	−0.33	52
TICS Excessive Demands at Work	8.73 (4.71)	7.86 (5.75)	51	−0.40, 2.13	0.65 ***	1.37	50
TICS Social Overload	7.53 (4.12)	5.53 (4.22)	51	0.78, 3.22	0.46 ***	3.29 **	50
TICS Work Overload	13.73 (5.86)	12.73 (7.05)	51	−0.85, 2.85	0.49 ***	1.08	50
HADS Depression	5.17 (3.67)	6.21 (4.06)	53	−2.07, −0.01	0.54 ***	−2.02 *	52
PANAS Positive	27.44 (8.51)	24.35 (8.87)	52	1.49, 4.70	0.78 ***	3.88 ***	51
FNR	12.38 (3.43)	12.49 (3.65)	53	−1.03, 0.81	0.56 ***	−0.25	52
Self-distraction	3.06 (1.58)	4.24 (1.41)	54	−1.77, −0.60	−0.02	−4.08 ***	53
Active coping	3.09 (1.44)	2.89 (1.56)	54	−0.33, 0.73	0.16	0.77	53
Denial	0.69 (1.15)	0.81 (1.20)	54	−0.50, 0.24	0.33 *	−0.70	53
Substance use	1.13 (1.83)	0.48 (1.09)	54	0.17, 1.12	0.38 **	2.74 **	53
Emotional support	2.94 (1.70)	2.87 (1.58)	54	−0.53, 0.68	0.09	0.25	53
Instrumental support	3.04 (1.68)	2.59 (1.54)	54	−0.18, 1.07	−0.01	1.43	53
Behavioral disengagement	1.30 (1.41)	1.02 (1.21)	54	−0.20, 0.75	0.12	1.17	53
Venting	2.13 (1.30)	2.17 (1.34)	54	−0.47, 0.40	0.28 *	−0.17	53
Positive reframe	3.09 (1.75)	3.54 (1.65)	54	−0.98, 0.09	0.34 *	−1.67	53
Planning	3.13 (1.66)	2.63 (1.58)	54	−0.08, 1.08	0.16	1.74	53
Humor	2.04 (1.65)	2.39 (1.51)	54	−0.78, 0.07	0.52 ***	−1.66	53
Acceptance	3.19 (1.39)	4.02 (1.30)	54	−1.24, −0.42	0.38 **	−4.08 ***	53
Religion	2.19 (1.91)	1.89 (1.87)	54	−0.15, 0.75	0.62 ***	1.32	53
Self-blame	2.46 (1.70)	1.30 (1.24)	54	0.68, 1.65	0.29 *	4.79 ***	53

*Note*. * *p* < 0.05, ** *p* < 0.01, *** *p* ≤ 0.001. *Abbreviations*: M, Mean. SD, Standard Deviation. CI, Confidence Interval. r, paired samples correlation. IDAS-II, Inventory of Depression and Anxiety Symptoms-Expanded Version. BIS, Behavioral Inhibition Scale. PSWQ, Penn State Worry Questionnaire. TICS, Trier Inventory of Chronic Stress. HADS, Hospital Anxiety and Depression Scale. PANAS, Positive and Negative Affect Schedule. FNR, Frustrative Non-Reward.

## Data Availability

The data presented in this study are available on request from the corresponding author.
